# The Role of Carbon Dioxide in the Rat Acute Stroke Penumbra

**DOI:** 10.3389/fdgth.2021.824334

**Published:** 2022-02-04

**Authors:** Leonard L. Yeo, Fabian Arnberg, Arvin Chireh, Vijay Sharma, Benjamin Tan, Vamsi Gontu, Philip Little, Staffan Holmin

**Affiliations:** ^1^Departments of Neuroradiology, Karolinska University Hospital, Stockholm, Sweden; ^2^Department of Clinical Neuroscience, Karolinska Institutet, Stockholm, Sweden; ^3^Division of Neurology, Department of Medicine, National University Health System, Singapore, Singapore; ^4^Yong Loo Lin School of Medicine, National University of Singapore, Singapore, Singapore

**Keywords:** carbon dioxide, acute stroke, arterial spin label (ASL) MRI, Rat—brain, penumbra, middle cerebral arterial occlusion

## Abstract

**Purpose:**

The vasodilatory response to inhaled CO_2_ occurs in the acute stroke ischemic penumbra and may be a potential therapeutic modality.

**Methods:**

Twenty-two Sprague-Dawley rats were subjected to 90-min occlusion of the M2 segment of the middle cerebral artery (M2CAO) by endovascular technique. The animals were administered different C02 concentrations and scanned serially with 9.4 T MRI. Infarct tissue was determined by diffusion-weighted imaging (DWI) and hypoperfused tissue was determined by arterial spin labeling (PWI).

**Results:**

4 animals were administered room air (RA)+ 6% CO_2_ (group 1), 6 animals RA+12% CO_2_ (Group 2) and 4 animals only RA (group 3). In the rats with CO_2_ administered (groups 1 and 2), the DWI lesion to cerebral hypoperfusion volume ratio (SD) at pre-CO_2_ administration, was 0.145(0.168), which increased to 0.708(0.731) during CO_2_ administration and reduced to 0.533(0.527) post-CO_2_ administration. In 9 of 10 rats the hypoperfused volume decreased when CO_2_ was administered. When CO_2_ was stopped the hypoperfused volume became larger again. Administration of RA+12% CO_2_ (Group 2) decreased the volume of CBF hypoperfusion significantly compared to the control group (95%CI: 0.084 ± 0.0213, *p* = 0.004).

**Conclusion:**

Inhaled CO_2_ appears to reduce the size of the hypoperfused tissue volume during acute stroke and may be a potential modality for treatment of acute ischemic stroke. These findings will nonetheless need to be validated in a larger cohort in other centers.

## Introduction

Stroke is the second most common cause of death worldwide and the major cause of disability (1). During an acute ischemic stroke, the leptomeningeal collateral circulation keeps the ischemic penumbra in the brain from succumbing to infarction (2). Studies have shown that in some patients, the ischemic penumbra can last hours or even days before it becomes infarcted (3). The discovery of factors that can affect the perfusion by modulating the leptomeningeal collaterals and sustaining the penumbra will allow more patients to be treated by recanalization therapies.

Carbon dioxide (CO_2_) is one of the strongest natural vasodilators. Cerebral blood flow (CBF) changes in concordance with changes in the partial pressure of CO_2_ (PaCO_2_) within the range of 20–80 mm Hg (3). All cerebral vessels respond to alterations in CO_2_, but hypercapnia has been shown to dilate smaller arterioles more than larger ones (3, 4). Nonetheless in a hyperacute stroke, the reactivity to CO_2_ could be different. In swine experiments, hypocapnia was found to be associated with reduced cerebral blood flow and could increase the ischemic penumbra (5). Conversely, in humans increased CO_2_ has been associated with the “reversed Robin Hood syndrome” which is a steal phenomenon linked with worse clinical outcomes (6). In humans with sleep apnoea syndrome, non-invasive positive pressure ventilation to increase ventilation of CO_2_ has been used as an adjunctive treatment during acute stroke (7).

The reactivity of blood vessels to CO_2_ in stroke is well described (8–13) and there are several studies which have looked at the effects of high levels of CO_2_ on rodents with middle cerebral artery ligation occlusion (MCAO) which has shown a protective clinical effect (14, 15). However the use of CO_2_ as an adjunctive treatment has not gained widespread use (7). This is partially due to the worry of increased CO_2_ acidosis causing worse outcomes in the context of acute ischemic stroke, as well as poor understanding of the underlying physiological changes in cerebral perfusion associated with CO_2_ which can translate into clinical outcomes (16, 17).

We seek to bridge this gap and we hypothesize that CO_2_ will affect the leptomeningeal collateral circulation and can be used to modulate cerebral perfusion in acute ischemic stroke. Administering CO_2_ could either help in maintaining a penumbral tissue state or it may worsen the ischemia by stealing blood away from affected area. We therefore performed an experiment where CO_2_ was administered in different concentrations in rats who had acute stroke to determine if it would change the volume of the ischemic penumbra.

## Methods

All animal handling and experiments were conducted according to the guidelines provided by the Animal Welfare Board at Karolinska Institute, Stockholm, Sweden. This study was approved by the Stockholm Northern Regional Ethical Committee (Ethical approval #N4/15). Animals in research is regulated by a common law EU framework (Directive 2010/63/ EU) and the experiments were conducted in compliance with the Animal Research: Reporting *In-Vivo* Experiments (ARRIVE) guidelines.

Twenty-two Male Sprague-Dawley rats (350–400 grams, approximately 14 weeks age, Scanbur B&K, Sollentuna, Sweden) were maintained on 12:12 light:dark cycle (lights on at 0900 h) and provided food and water *ad libitum*. Approximately during anesthesia, animals were kept normothermic by means of a rectal thermistor coupled with a heating pad.

Anesthesia was initially induced using 4% isoflurane mixed with 100% O2 and subsequently maintained at 2% isoflurane concentration in an air:oxygen mixture (7:3), which was later switched to a sub-cutaneous medetomidine infusion (500 ug over 1 h) before occluding the distal middle cerebral artery. The switch to medetomidine was performed to prevent vasodilatory effects of isoflurane from affecting the CO_2_ dilatory effects. Anesthesia was confirmed by a reduction in the breathing rate and the absence of a withdrawal reflex in response to tail pinch. Animal vital signs were monitored throughout the surgical procedure and a piezo electric force transducer mounted on the animal's thorax to determine the respiratory rate and amplitude of respiration while in the MRI scanner.

All animals were subjected to occlusion of the distal middle cerebral artery as described previously, (M2CAO) (18). Briefly, an incision was made in the tail artery and a 1.2F Magic hydrophilic microcatheter (BALT, Irvine, CA) was advanced to the proximal descending aorta carrying a 0.007-inch Hybrid microwire (Balt Extrusion, Montmorency, France). Subsequently, the tip of the microwire was navigated to, and positioned in the base of the internal carotid artery to allow blood flow past. The rats were then switched to the aforementioned sub-cutaneous medetomidine (Domitor®, Pfizer) infusion and the isoflurane was allowed to wash out for approximately 30 min, during which time the animals were placed into a restrainer consisting of a multiconcentric, acrylic head and body holder with built-in dual coil radiofrequency electronics fitted with a ventilation tube for gas inhalation. This helped to maintain spatial consistency between examinations and to reduce motion artifacts. The guidewire was then advanced to the distal middle cerebral artery causing an M2 occlusion (M2CAO). Next, the animal was transferred to a 9.4-T MRI scanner, where a continuous flow of heated air was used to maintain animal body temperature during the examination.

## Study Design

**Figure 1:**
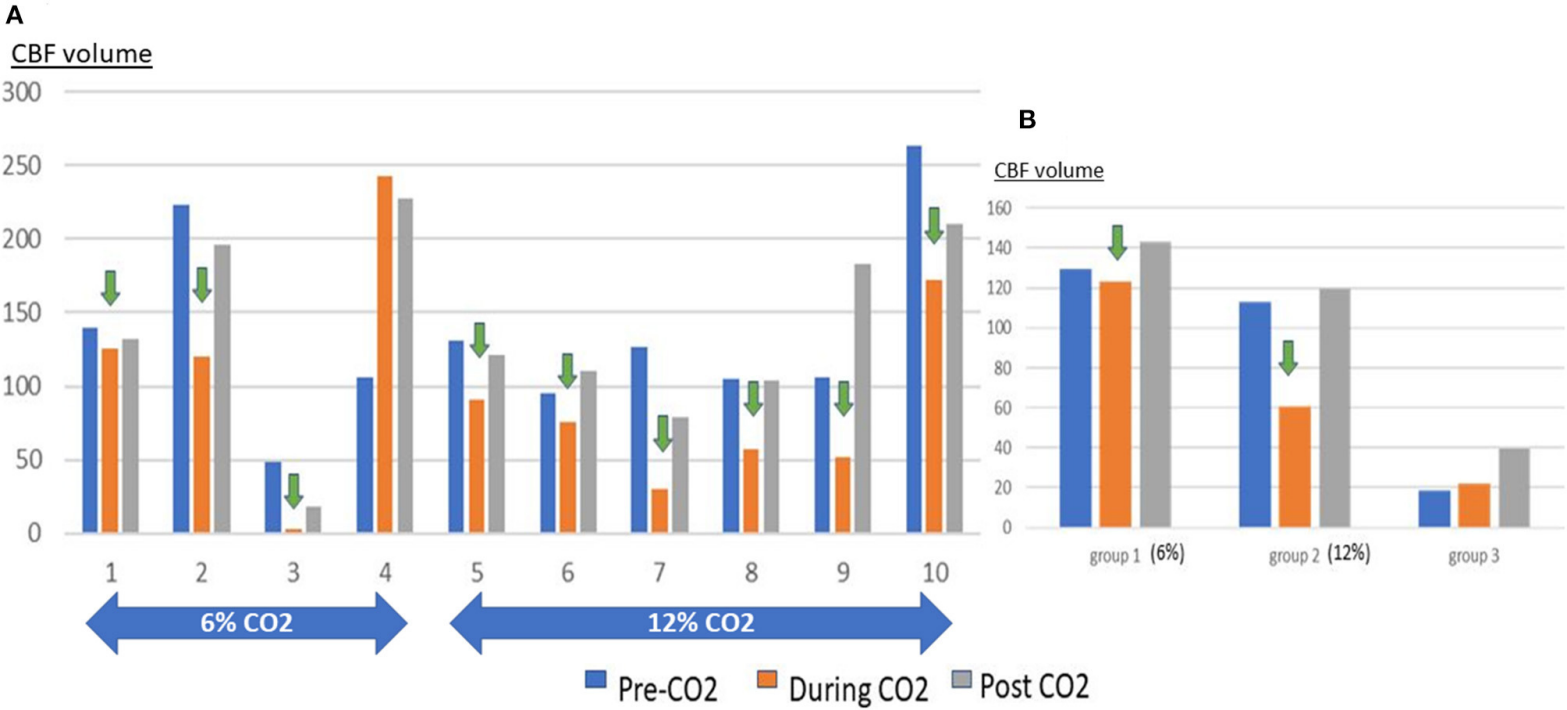
Graphs showing the volume of CBF hypoperfusion at pre-CO_2_, CO_2_ administration and post-CO_2_ time points. **(A)** shows the individual rats, Note the reduction of CBF hypoperfusion with CO_2_ administration and rebound when it is stopped. **(B)** shows the results in group 1 (6% CO_2_), group 2 (12% CO_2_) and group 3 (Control). Note the increased CBF hypoperfusion with 12% CO_2_ compared to 6% CO_2_.

The animals were divided into three groups, those subjected to either pre-mixed air with 6% CO_2_ (group 1), air with 12% CO_2_ (Group 2) or room air (without supplemental CO_2_), (Group 3). The animals were not randomized for this study. The mole fraction of O2 in these gas mixtures was maintained at 21% by reducing the fraction of nitrogen. After the M2CAO was induced as described, the rats underwent MRI scanning. After the 1st imaging sequence, the pre-mixed CO_2_ gas was turned on and the rats underwent a second series of MRI scans. The pre-mixed CO_2_ gas was then turned off and the rats underwent a final series of MRI scans. At the end of the experiment, the rats were euthanized through decapitation while in deep anesthesia and both hemispheres of the brain were harvested (Figure 1).

## Magnetic Resonance Imaging

The animals were transferred to the MRI scanner within 5 min of the placement of the microwire to occlude the M2 branch of the MCA. Diffusion-weighted imaging and arterial spin labeling was obtained with three-dimensional volumetric data of the entire brain. This was performed with a MRI comprised of a horizontal 9.4T magnet (Varian, Yarnton, United Kingdom), with a 120 mm inner diameter gradient system and a maximum gradient strength of 600 mT/m. The T2 weighted sequences were acquired with 1 mm thick slices in the coronal plane (13 slices), using multi-slice three-shot spin-echo echo-planar imaging. Using a field of view (FOV) of 32 x 32 mm, repetition time (TR) of 3 s, echo time (TE) of 25 ms and matrix size of 96 x 96 zero filled to 128 x 128, resulted in an in-plane resolution of 256 μm (18, 19). Diffusion weighted images were acquired with a diffusion gradient duration (Δ): 2.3 ms, diffusion gradient separation (δ): 6.5 ms. Diffusion sensitizing gradients were applied along 12 directions with a *b* value = 1,000 s/mm^2^ and a control image collected twice with the diffusion sensitizing factor *b* = 0. Apparent diffusion coefficient (ADC) maps (mm^2^/s) were then obtained from this data (18, 19).

For perfusion sequences, an actively tuned arterial spin labeling (ASL) surface coil (Rapid Biomedical GmbH, WuÈrzburg-Rimpar, Germany) was positioned under the neck of the animal. In order to have one coil tuned to the Larmor frequency at a time, the coils were actively decoupled through a pin diode driver with three independent channels (Rapid Biomedical GmbH, Wurzburg-Rimpar, Germany). The localized excitation of the neck coil did not extend to the brain, and magnetization transfer could safely be ignored with the actively decoupled three-coil configuration. PWI was acquired with continuous ASL and by single-shot gradient echo planar imaging by applying an off-resonance radio frequency power to the ASL coil simultaneously with a 1 Gauss/cm gradient during TR. The specifications for TR were: 6 s, TE: 10.2 ms, with the FOV and resolution were similar to the T2 weighted sequence. Tagged and non-tagged control images was acquired for each slice and there were 25 repetitions per slice. The labeling plane was located 24 mm up from the center of the applied slice package. This corresponds to 7 kHz off-resonance for the slice nearest to the labeling plane and increasing by 0.4 kHz for each slice. The RF power of the tag coil is put at zero for the control image (18, 19). For the perfusion images, the configuration and position of the slice package was the same as the DWI and T2 weighted sequence. From this data, the cerebral blood flow (CBF) maps (ml/g/min) were calculated. VnmrJ (Agilent Technologies, Palo Alto, CA, USA), ImageJ (National Institutes of Health, Maryland, USA), and OsiriX (OsiriX Foundation, Geneva, Switzerland) software were used for MRI data processing (18, 19). Each MRI scan took 20 min with the optimized protocol and to keep the duration uniform between experiments, the animal was not moved between each of the 3 scans per animal (without CO_2_, with CO_2_ administered and with CO_2_ stopped) (Figure 1).

**Figure 2:**
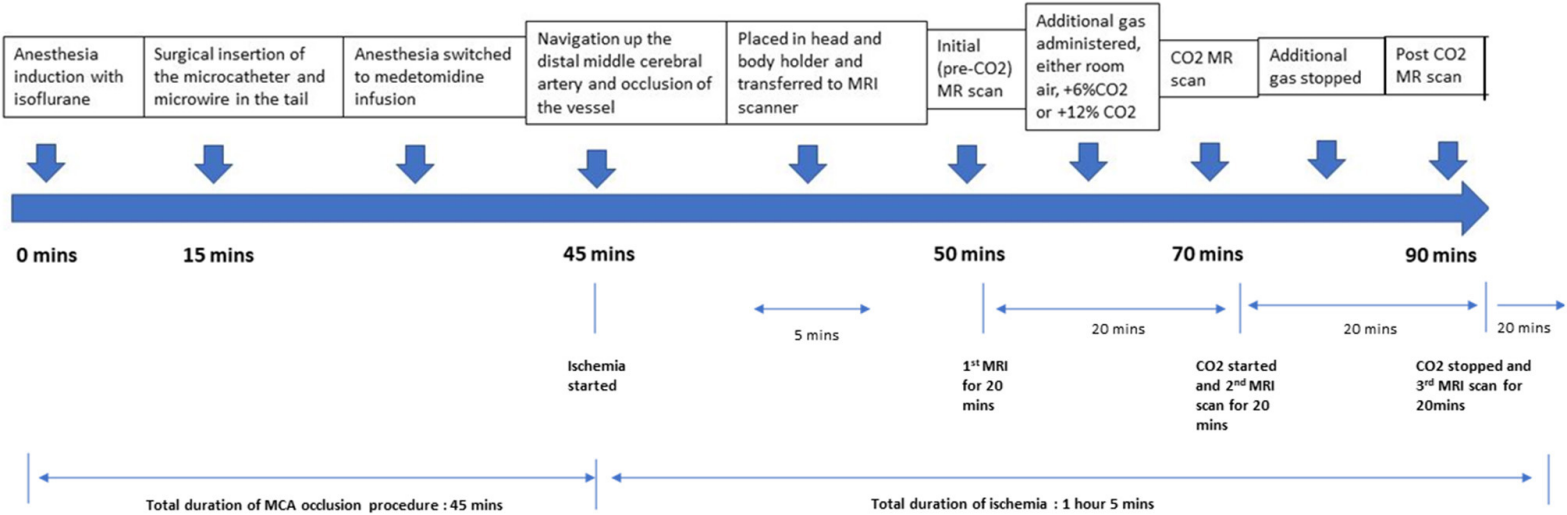
Timeline of the experiment, with approximate timing of each stage of the experiment below.

Regions of interest (ROI) segmentation was done with ImageJ and ITK-SNAP software, during which the assessor was blinded to which group the animals were from. Each image was visually inspected, and individual ROIs were manually traced areas with decreased ADC to delineate the ischemic core. ROIs were similarly manually traced around areas with reduced CBF seen on the ASL maps (Figure 2).

## Statistics

We present the numerical variables as mean and standard deviation (SD). Analysis of changes in volumes of cerebral perfusion at different CO_2_ concentrations were tested by using 2-sample *t-*test or Mann-Whitney *U* test where applicable. Associations were presented as odds ratios (OR) with corresponding 95% confidence intervals (CI). Statistical significance was defined as *p* < 0.05. Statistical analyses were performed using the Statistical Package for Social Sciences (SPSS) version 21.

## Results

An initial total of 22 animals had the M2CAO stroke procedure performed. 9 rats with 6% CO_2_ administered (group 1), 9 rats with 12% CO_2_ (Group 2) and 4 control rats without additional CO_2_ (group 3). Three animals in Group 1 and one animal in group 2 were excluded due to subarachnoid hemorrhage secondary to perforation of the MCA. One animal from group 2 was excluded due to absence of infarct on MRI. Two animals from group 1 and 1 animal from Group 2 died before transfer to the MRI scanner. In Rat 1 (from group 1) and control 2 (from group 3), the MRI scanner technical malfunction resulted in data loss of the DWI images during CO_2_ administration and post-CO_2_ administration. The post-CO_2_ administration DWI and ASL images were also lost for control 2 and control 4. Finally, Data were tabulated for each of the groups; room air + 6% CO_2_ (group1, *n* = 4), inhaling room air +12% CO_2_ (group 2, *n* = 6) and inhaling room air with no additional CO_2_ (control group, *n* = 4). All images were viewed and assessed for motion artifacts (Table 1).

**Table 1:** Ipsilateral ratio of diffusion weighted imaging (DWI) volume and arterial spin labeling hypoperfusion (CBHF) volume divided by ipsilateral hemispheric brain volume, at various time points and in the three groups.

**S/N**	**Additional CO_2_ concentration**	**Pre-CO_2_ DWI**	**CO_2_ DWI/**	**Post-CO_2_ DWI**	**Pre-CO_2_ CBFH**	**CO_2_ CBFH**	**Post-CO_2_ CBFH**	**pre-CO_2_ DWI/CBFH**	**CO_2_ DWI/CBFH**	**Post-CO_2_ DWI/CBFH**
**Rat 1**	6%	0.005	Not done	Not done	0.198	0.179	0.187	0.026	Not done	Not done
**Rat 2**	6%	0.082	0.086	0.092	0.319	0.172	0.281	0.258	0.497	0.327
**Rat 3**	6%	0.007	0.003	0.006	0.062	0.004	0.023	0.107	0.795	0.256
**Rat 4**	6%	0.081	0.144	0.139	0.143	0.329	0.307	0.568	0.437	0.453
**Rat 5**	12%	0.024	0.009	0.027	0.172	0.118	0.158	0.142	0.077	0.168
**Rat 6**	12%	0.005	0.004	0.013	0.127	0.101	0.148	0.042	0.037	0.090
**Rat 7**	12%	0.002	0.031	0.061	0.173	0.042	0.109	0.011	0.736	0.558
**Rat 8**	12%	0.014	0.201	0.276	0.151	0.081	0.149	0.095	2.467	1.848
**Rat 9**	12%	0.004	0.066	0.098	0.139	0.067	0.240	0.028	0.979	0.410
**Rat 10**	12%	0.063	0.083	0.199	0.360	0.236	0.288	0.175	0.350	0.689
**Control 1**	Nil	0.123	0.098	0.104	0.074	0.075	0.126	1.655	1.310	0.821
**Control 2**	Nil	0.005	Not done	Not done	0.002	0.016	Not done	2.125	Not done	Not done
**Control 3**	Nil	0.023	0.008	0.010	0.007	0.018	0.027	3.368	0.443	0.377
**Control 4**	Nil	0.002	0.002	Not done	0.014	0.006	Not done	0.163	0.418	Not done

*DWI, diffusion weighted imaging; CO_2_, carbon dioxide; CBFH, cerebral blood flow hypoperfusion; DWI/CBF, diffusion weighted volume divided by cerebral blood flow hypoperfusion volume*.

In rats administered CO_2_ (Group 1 and 2 combined), the mean volume of infarcted tissue as measured by the ratio of the ADC lesion to the hemispheric volume (mm3±SD) was 0.028 (0.033), 0.069 (0.068) and 0.101 (0.09) for pre-CO_2_, during CO_2_ and post-CO_2_ administration, respectively. While in the control group the ADC lesions were 0.039 (0.057), 0.036 (0.536) and 0.057 (0.066) for the corresponding sequences. The mean volume of hypoperfused tissue as measured by ASL (mm^3^ ± SD) for the whole cohort of rats administered CO_2_ (group 1 and 2 combined) was 0.184 (0.089), 0.133 (0.098) and 0.189 (0.090) for pre-CO_2_, during CO_2_ and post-CO_2_ administration, respectively. While in the control group the mean volume of hypoperfused tissue was 0.024 (0.034), 0.029 (0.313) and 0.767 (0.069) for the corresponding sequences (Table 1).

**Figure 3:**
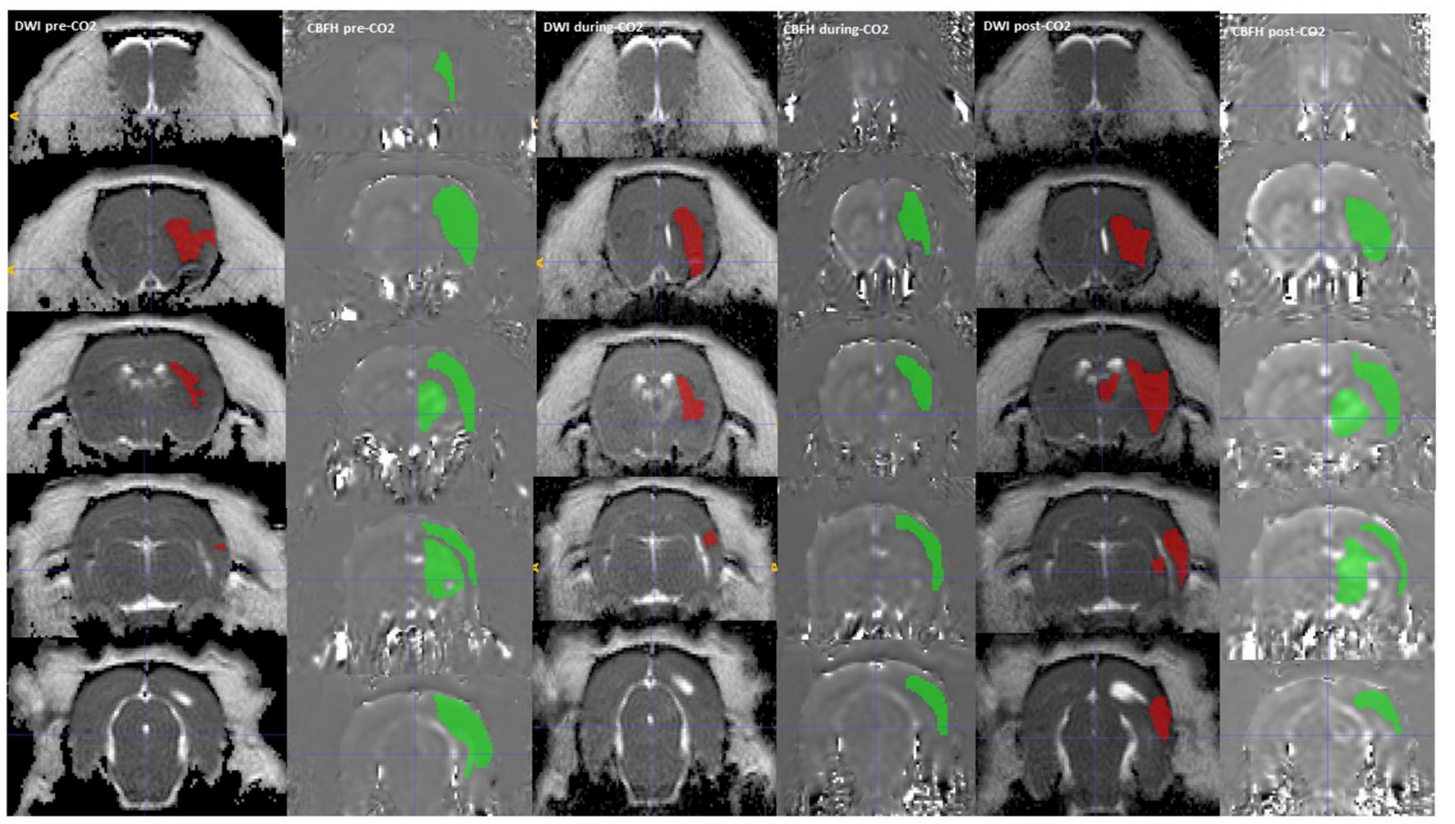
A DWI (Green) and CBF hypoperfusion, CBFH (Red) region of interest drawn onto the corresponding MRI sequences, showing a reduction in CBF hypoperfusion volume with administration of CO_2_. The CBF hypoperfusion volume reverses and increases with stoppage of CO_2_.

In the rats administered CO_2_ (Groups 1 and 2 combined) the DWI lesion to cerebral hypoperfusion volume ratio (SD) was 0.145 (0.168) at pre-CO_2_ administration, this increased to 0.708 (0.731) during CO_2_ administration and subsequently reduced to 0.533 (0.527) post-CO_2_ administration. In 9 out of 10 rats the volume of the hypoperfused tissue decreased when CO_2_ was administered but this decrease reversed when the CO_2_ was discontinued (Figure 3). The mean decrease in CBF hypoperfusion was 0.051 (0.094) for all rats with CO_2_, 0.079 (0.412) for rats breathing room air + 12% CO_2_ and 0.0094 (0.141) for rats breathing room air + 6% CO_2_. Only administration of room air + 12% CO_2_ decreased the volume of CBF hypoperfusion significantly when compared to the control group (95%CI: 0.034–0.133, *p* = 0.004) (Table 2). Rat 4 from group 1 did not have a reduction in CBF hypoperfusion with administration of CO_2_, this is likely due to the large infarct size which involved the hypothalamic region, which caused hyperthermia throughout the experiment (Supplementary Figure 1).

**Table 2:** Changes in cerebral blood flow hypoperfusion (CBFH)/hemisphere volume: 6 and 12% carbon dioxide (CO_2_) groups versus control groups.

	**Control group Mean (SD)**	**6% CO_2_ group** **Mean (SD)**	**95% CI, P-value**	**12% CO_2_ group** **Mean (SD)**	**95%, *P*-value**
**Pre CO**_**2**_ **vs. during CO**_**2**_	−0.004 (0.009)	0.079 (0.412)	−1.59 to 0.186, *P* = 0.853	0.0094 (0.141)	0.034 to 0.133, *P* = 0.004
**During CO**_**2**_ **vs. post CO**_**2**_	0.03 (0.029)	0.074 (0.049)	−0.491 to 0.137, *P* = 0.291	0.283 (0.056)	−0.124 to 0.120, *P* = 0.966

## Discussion

Our study shows that CO_2_ can reduce the volume of CBF hypoperfusion during acute ischemic stroke in rats and was seen in 90% of our experimental cohort. There was also a strong suggestion of a dose dependent response where the 12% CO_2_ group showed a significant decrease in CBF hypoperfusion compared to controls, but not the 6% CO_2_ group.

Stroke is the fifth cause of death in the USA and the leading medical cause of acquired adult disability worldwide (20, 21). The ischemic penumbra, the area of ischemic brain tissue surrounding the infarcted core, is potentially salvageable if an appropriate treatment is administered within a specified therapeutic window. (22) There are two major approaches to treat acute ischemic stroke: neuroprotection and reperfusion. Reperfusion by thrombolysis and/or interventional thrombectomy has changed the treatment paradigm owing to their high efficacy, however, only a limited number of patients benefit from reperfusion therapies due mainly to time constraints. Recent efforts have been undertaken to identify acute therapies which can potentially prolong the deterioration of the ischemic penumbra, and to better understand the mechanisms by which this tissue is irreversibly damaged. However an ideal neuroprotective agent for acute ischemic stroke remains elusive (22–25).

CO_2_ is a strong vasodilator in the brain and can affect the cerebral blood flow with commensurate changes of up to 4% per change in PaCO_2_ in humans (26). In the presence of carbonic anhydrase, elevated levels of CO_2_ form carbonic acid in the blood, creating an acidic environment that enhances the vasodilatory effects of adenosine and increases potassium ion conductance across vascular smooth muscles. Cerebral arterial smooth muscles are sensitive to the partial pressure of CO_2_ in the blood, and this response appears to be mainly modulated by extracellular fluid pH. The result is a dilation of blood vessels with decreased resistance and increased blood flow (27–29). Although all cerebral vessels appear to respond to changes in PaCO_2_, the vasodilatory effects appears to be more in smaller arterioles than larger ones (26).

This is the first animal acute stroke study that shows the effects of CO_2_ in reducing the volume of hypoperfused tissue using serial perfusion imaging, that we are aware of. In this study, the DWI volume steadily increased in the rats administered CO_2_, which is what is normally expected in the evolution of stroke, but the DWI/hypoperfused volume ratio fluctuated due to a reduction in the hypoperfused volume when CO_2_ was administered. Importantly, when the CO_2_ administration was stopped, there was a rebound in the volume of hypoperfusion volume showing that the reduction was indeed dependent on the CO_2_ and not on other factors. This could potentially mean that the ischemic penumbra in acute stroke patients could be kept viable with CO_2_ administration until they received re-canalization therapy. We also observed a strong indication of a dose dependent response to the CO_2_ concentration. While there are few studies on CO_2_ administration in ischemic stroke, a study reported a reduction in CBF in rats when CO_2_ concentration was decreased, and this was postulated to potentially worsen the area of infarction (30). This study corroborates our findings.

Only Rat 4 in our study did not have a reduction in the CBF hypoperfusion volume with 12% CO_2_ administration. Rat 4 had a large hemispherical infarct involving the hypothalamus, resulting in the high temperature ranging from 38.9 to 41° that the animal sustained throughout the experiment and consequently, affected the vasodilation. Although this is a single animal and may not be generalizable, nonetheless it suggests that certain types of stroke may not respond to CO_2_ administration. Interestingly, rat 10 also had a similar sized stroke but the hypothalamic area was spared, and the rat showed good response to 12% CO_2_ with a reduction in CBF hypoperfusion which reversed when the CO_2_ was stopped.

Previous experiments involving various treatment modalities for preserving the ischemic penumbra in rats have not been very effective in translating to humans. This may be due to the difference in cerebral physiology between humans and rats. The most widely used model, for focal cerebral ischemia, is the intra-arterial suture occlusion of the middle cerebral artery (MCAO) in rats (31, 32) where a monofilament is inserted into a transected external carotid artery and extended to occlude the origin of the middle cerebral artery. The main issue with the MCAO model is the limitation of blood flow to the anterior and posterior cerebral artery resulting in extensive ischemic infarcts mimicking a large hemispheric stroke. The hemispheric occlusion inhibits collateral flow from adjacent vessels, rendering it a suboptimal model for studying the effects of the collateral circulation in stroke (33, 34). Secondly, in rats, the posterior cerebral artery, arising from the internal carotid terminus, is the main tributary to the thalamus, hypothalamus, hippocampus, and substantia nigra (35–37). Infarction in the above important regions lead to a variable level of increased body temperature, disturbed water homeostasis, and severe paresis, which introduces bias in experimental studies. Human strokes tend to encompass 4.5–14% of the volume of the ipsilateral hemisphere compared with 21–45% in the MCAO rat models, and therefore in combination with the limited pial collateral circulation with the MCAO model, results obtained through this model tend to not translate to human infarcts (33, 34).

In the present study, we used a recently described model for inducing a small focal cortical infarction that preserves collateral flow (19). Selective M2 occlusion does not disrupt collateral blood flow to the MCA from the anterior and the posterior cerebral arteries, thus producing ischemic stroke which are more similar in both size and regional blood flow to those commonly found in human patients. In a previous study by our group using the same distal MCA technique, we noted an initial rapid occurrence of both cytotoxic and vasogenic edema within the ischemic core. Although this core rapidly undergoes infarction, the subsequent spread of infarction in the initial hypoperfused area occurs at a slower pace (19), similar to a human clinically in an ischemic stroke, and this was likely due to the preserved collateral blood flow of the M2CAO model. The preservation of collateral supply and gradual onset of ischemic changes in the M2CAO stroke model are important in our study, particularly as we look for cortical CBF changes due to CO_2_ administration.

We acknowledge some limitations of the current study. Being an animal experiment with a small sample size has its inherent limitations and there was no randomization of the groups. Moreover, out of 22 rats, only 14 were usable for the analysis that may have introduced some bias. The other main limitation is the lack of vascular resistance measurements and endothelial or smooth muscle histology that could help elucidate the mechanism producing the differences, and the lack of validation of the infarct volume by histology. The stroke model is designed to create smaller distal M2 infarcts, however sub-cortical structures were sometimes be involved in the infarct. This is a problem inherent in the model, nonetheless, the volume of infarct is smaller than that seen in an MCAO model. We also recognize that CBF hypoperfusion is a dynamic variable which may not directly reflect the evolution of ischemia in a linear fashion and this may be affected by the collateral circulation in the animals. There was a larger CBF hypoperfusion volume and somewhat more rapid lesion growth in animals administered with CO_2_, compared to the control group. This may be due to combination of smaller stroke sizes induced in the control group and the limited sample size of the control group. The control group may also have had better collateral circulation. Finally, we are not suggesting imminent use of CO_2_ as an acute treatment, as further validation studies are warranted, followed by appropriate human stroke trials studies.

## Conclusion

Carbon dioxide in acute stroke appears to improve the cerebral blood flow and reduce the size of the perfusion defect. This is a cheap and potentially useful therapeutic modality with widespread availability which could be used to help stabilize the collaterals and maintain the penumbra until recanalization therapy is performed.

## Data Availability Statement

The original contributions presented in the study are included in the article/Supplementary Material, further inquiries can be directed to the corresponding author/s.

## Ethics Statement

This study was approved by the Stockholm Northern Regional Ethical Committee (Ethical approval #N4/15).

## Author Contributions

LY designed the experiment and carried it out, analyzed and interpreted the data and wrote the manuscript. FA and SH helped to design the experiment and edited the manuscript. AC and VG helped designed the experiment and help to carry it out and edit the manuscript. VS and BT helped to design the experiment and edit the manuscript. PL helped to design the experiment and carry it out, analyse the data and edited the manuscript. All authors contributed to the article and approved the submitted version.

## Funding

LY was funded by an NMRC Research Training Fellowship (NMRC/FLWSHP/043/2017) for this study.

## Conflict of Interest

The authors declare that the research was conducted in the absence of any commercial or financial relationships that could be construed as a potential conflict of interest.

## Publisher's Note

All claims expressed in this article are solely those of the authors and do not necessarily represent those of their affiliated organizations, or those of the publisher, the editors and the reviewers. Any product that may be evaluated in this article, or claim that may be made by its manufacturer, is not guaranteed or endorsed by the publisher.

## Supplementary Material

Supplementary Figure 1 Rat 4 which did not show a reduction in ASL lesion volume with CO_2_ administration. The MRI sequences show a large infarct with involvement of the hypothalamic region (yellow arrow) that resulted in hyperthermia of the animal throughout the experiment.
