# Supplementary material for: The Role of Carbon Dioxide in the Rat Acute Stroke Penumbra
Source: Front Digit Health. 2022 Feb 4;3:824334. doi: 10.3389/fdgth.2021.824334 (PMC8854855; doi:10.3389/fdgth.2021.824334)

Supplementary Figure 1:

***The role of carbon dioxide in the rat acute stroke penumbra***

***A potential new modality for stroke treatment***

Leonard LL Yeo^1-4^, MBBS, Fabian Arnberg^1,2^, MD PhD, Arvin Chireh^2^, MD PhD, Vijay K Sharma^3,4^, MD, Benjamin Tan^3,4^, MBBS, Vamsi Gontu^1,2^, MD, Philip Little^1,2^, MD PhD, Staffan Holmin^1,2^, MD PhD.

^1^ Departments of Neuroradiology, Karolinska University Hospital, Stockholm, Sweden

^2^ Department of Clinical Neuroscience, Karolinska Institutet, Stockholm, Sweden

^3^ Division of Neurology, Department of Medicine, National University Health System, Singapore

^4^Yong Loo Lin school of medicine, National University of Singapore

Legend for Supplementary Figure 1: Rat 4 which did not show a reduction in arterial spin labelling (ASL) lesion volume with CO2 administration. The MRI sequences show a large infarct with involvement of the hypothalamic region (yellow arrow) that resulted in hyperthermia of the animal throughout the experiment. This animal may not have responded to CO2 administration due to the hyperthermia.


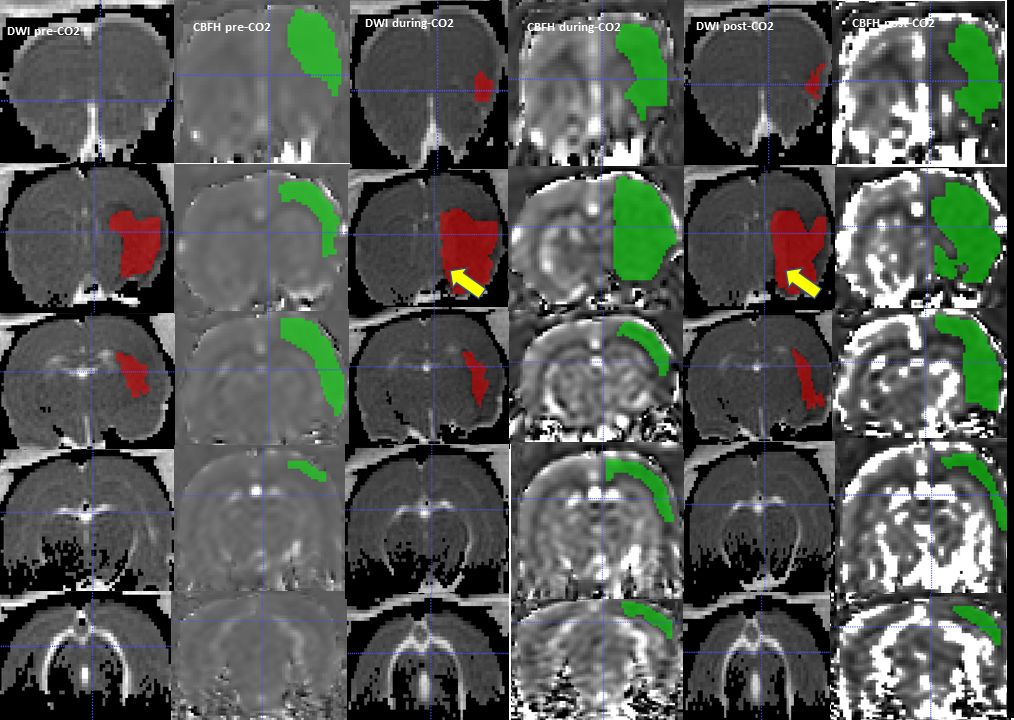

Supplement: Supplementary Figure 1 — Rat 4 which did not show a reduction in ASL lesion volume with CO2 administration. The MRI sequences show a large infarct with involvement of the hypothalamic region (yellow arrow) that resulted in hyperthermia of the animal throughout the experiment. [file Data_Sheet_1.docx]
